# Deep Sedation for Pediatric Parotid Sialendoscopy in Juvenile Recurrent Parotitis

**DOI:** 10.3390/jcm10020276

**Published:** 2021-01-13

**Authors:** Pasquale Capaccio, Andrea Palermo, Paolo Lucchinelli, Tiziana Marchesi, Sara Torretta, Michele Gaffuri, Paola Marchisio, Lorenzo Pignataro

**Affiliations:** 1 Fondazione IRCCS Ca’ Granda Ospedale Maggiore Policlinico, 20100 Milan, Italy; pasquale.capaccio@unimi.it (P.C.); andrea.palermo@studenti.unimi.it (A.P.); paolo.lucchinelli@studenti.unimi.it (P.L.); tiziana.marchesi@policlinico.mi.it (T.M.); michele.gaffuri@policlinico.mi.it (M.G.); paola.marchisio@unimi.it (P.M.); lorenzo.pignataro@unimi.it (L.P.); 2 Department of Biomedical, Surgical and Dental Sciences, Università degli Studi di Milano, 20100 Milan, Italy; 3 Department of Clinical Sciences and Community Health, Università degli Studi di Milano, 20100 Milan, Italy; 4 Department of Pathophysiology and Transplantation, Università degli Studi di Milano, 20100 Milan, Italy

**Keywords:** juvenile recurrent parotitis, sialendoscopy, pediatric age, sedation

## Abstract

Sialendoscopy is a minimally invasive diagnostic and therapeutic tool for juvenile recurrent parotitis (JRP); the procedure is under general anesthesia, but local anesthesia has been used for sialendoscopy in children >8 years. Based on the experience in children with sedation for gastrointestinal endoscopy, we investigated the reliability and safety of deep sedation for sialendoscopy in JRP. Six children (3 females, 6–13 years) with episodes of parotid swelling underwent interventional (duct dilation and steroid irrigation) sialendoscopy with intravenous bolus of 1 mg/kg propofol and 1 mcg/kg fentanyl, and continuous infusion of 2 mg/kg/h propofol. Sialendoscopy under deep sedation was successfully performed in all the patients; the procedure was well tolerated, without any adverse effects. One event of full awakening was registered and promptly solved without needing to interrupt the procedure. Effectiveness of sialendoscopy under deep sedation was subjectively attested by high positive scores obtained at post-operative standardized questionnaires administered to the patients and their parents, and objectively by the lack of clinical recurrences during the follow-up. The combination of propofol and fentanyl seems to be a reliable and safe means of sedating children with JRP undergoing sialendoscopy.

## 1. Introduction

Sialendoscopy is developing not only as a valid diagnostic tool but as an effective and safe treatment option for Juvenile Recurrent Parotitis (JRP) [1,2] in combination with conservative pharmacological treatment (steroid and non-steroid anti-inflammatory drugs, sialogogues) and as an alternative to other interventional techniques such as parotidectomy [3,4,5,6]. Although these last options are still adopted, sialendoscopy seems to be preferred in terms of recurrence rate, reduction of cycles of antibiotics and pharmacological side effects; it consists of direct visualization of the parotid ductal system with a small-caliber semiflexible endoscope, usually combined with saline and/or corticosteroid irrigation.

The physiopatological pathway leading to parotid inflammation in JRP is not completely understood, although Ussmuller and Donath [7] showed, by histological specimens, that JRP is a mucosa-associated lymphoid tissue disorder with hyperplastic cells surrounding inflamed ducts; in this regard, duct steroid irrigation during sialendoscopy may have a synergic anti-inflammatory and immunoregulatory action. On the other hand, dental malocclusion with dysfunction of masticatory muscles (in particular, masseter muscles) and genetic factors may increase susceptibility to JRP [8]. Finally, sialendoscopy has a valid diagnostic value in depicting duct anomalies or inflammatory duct and intraluminal features (mucous plugs, variable duct vascularization, inflammatory granulation tissue, duct kinking) [6].

Pediatric sialendoscopy for JRP is mainly based on duct dilation and irrigation, but is generally performed under general anesthesia [1,2]; very few studies report on the use of sialendoscopy for children, mainly older than eight years [9,10]. The use of deep sedation is becoming increasingly popular for children undergoing upper gastrointestinal endoscopy; different therapeutic protocols are used and mainly based on the combination of drugs such as midazolam-ketamine or fentanyl-propofol [11,12]. The combination of fentanyl and propofol is associated with a faster recovery time and fewer side effects, although it is less comfortable with respect to the midazolam-ketamine combination [11].

Our aim was to evaluate the feasibility of using deep sedation for sialendoscopy in children with JRP, and to extrapolate information about the success of the sialendoscopic procedure, surgical and anesthesiologic complications, and patient discomfort with a dedicated questionnaire.

## 2. Materials and Methods

Six consecutive naive children (3 female; mean age 10, median age 11, range 6–13 years) with recurrent episodes of unilateral (four) or bilateral (two) parotid swelling (median elapsed time since onset: 2 years; median: 1–4 years) were enrolled in the study and underwent sialendoscopy under deep sedation at our Department of Otolaryngology and Head and Neck Surgery between November 2019 and February 2020.

Patients underwent an accurate clinical history, ear-nose-throat (ENT) objective evaluation by means of inspection and palpation of the oral cavity and major salivary glands. A high-resolution ultrasound and doppler US assessment [6] (Hitachi H21, 7.5 MHz, Hitachi High Technology Corp. Ltd., Tokyo, Japan) was done to show typical diffuse hypoechoic areas inside the parenchyma and diffuse heterogeneous parenchymal echoes corresponding to recurrent parotitis.

The presence of an acute phase of parotid inflammation was considered as an exclusion criterion. Acute phases were treated by means of oral betamethasone 0.2 mg/kg/die for 3–5 days; mechanical and gustatory stimulation by means of gland massage and oral intake of sialogogue (i.e., lemon juice) were considered additional treatments.

Patients and their parents were thoroughly informed about the procedure and gave their informed consent. The study was approved by our local institutional review board.

### Procedure

The procedure was performed in the operating theatre by an expert salivary surgeon and by a resident in training with anesthesiologic support. Patients were premedicated with intravenous administration of 15 mg/kg paracetamol and 1 mg/kg pantoprazole, prophylactic i.v. single dose 1 g amoxicillin was also given before the beginning of the anesthesiologic procedures. In a single case, clindamicine was preferred due to an amoxicillin adverse reaction history. Deep sedation was induced with an i.v. bolus injection of 1 mg/kg propofol and 1 mcg/kg fentanyl, and was maintained with continuous infusion of 2 mg/kg/h propofol. Another bolus of 1 mg/kg propofol was administered in a case of reversion to full awakening. 2 L/min oxygen was administered with nasal cannula for the whole duration of the procedure.

Stensen’s duct ostium was dilated with a blunt-tip lacrimal probe dilator (Karl Storz Co., Tuttlingen, Germany). Sialendoscopy was performed with a 0.8 mm semiflexible angled sialendoscope (Erlangen sialendoscope, Karl Storz Co., Tuttlingen, Germany), by proceeding along the duct system up to the tertiary ductal branches thanks to saline solution continuous irrigation; the whole ductal system was finally irrigated with 16 to 24 mg dexamethasone diluted in saline solution per gland. Sialendoscopy was considered concluded when all the branches were explored, pervious and free from mucous plugs. Immediate and three hours post-op pain was assessed with a 0 (no hurt at all) to 10 (hurts most) smiley scale. The patients were discharged 6 h after observation.

At the three months follow-up, two questionnaires were sent by mail to assess improvement and patient’s opinions about the procedure. The first, adapted from Gillespie et al. [13], investigated the perceived symptoms and satisfaction after sialendoscopy; the second, from Iacobucci et al. [14], evaluated the parents and patient comfort with deep sedation. A telemedicine assessment was subsequently performed in December 2020 by means of a telephone call in order to register the number of recurrences, avoiding unnecessary access to the hospital during the COVID-19 pandemic. In particular, parents were asked to refer whether the children had experienced any further swelling of the treated gland or complained about the development of any new onset salivary-gland related symptoms (i.e., pain, skin redness, medical evaluation, needing for any medical treatment).

## 3. Results

Pediatric interventional sialendoscopy under deep sedation was successfully performed in all the patients. During sedation, spontaneous ventilation was constantly maintained. No events of major desaturation were registered. Only one event of full awakening was registered (patient n. 3), and promptly corrected by the anesthesiologist without needing to interrupt the procedure. Deep sedation was apparently well tolerated, without reports of any adverse effect during the observation period.

A bilateral parotid sialendoscopy was done in two patients while in the remaining patients sialendoscopy involved one gland (two left parotid glands). The median duration of sialendoscopy was 10 min per gland (range 10–13) and the median duration of the full procedure was 30 min (range 27–38). Clinical and sialendoscopic data are described in Table 1.

No major or minor complications were encountered except for mild swelling of glands persisting for a few hours after the procedure. The average immediate post-operative pain was 3.8/10 (range 0–8); three hours after the procedure, the average pain dropped to 1.6/10 (range 0–6).

**Figure 1:**
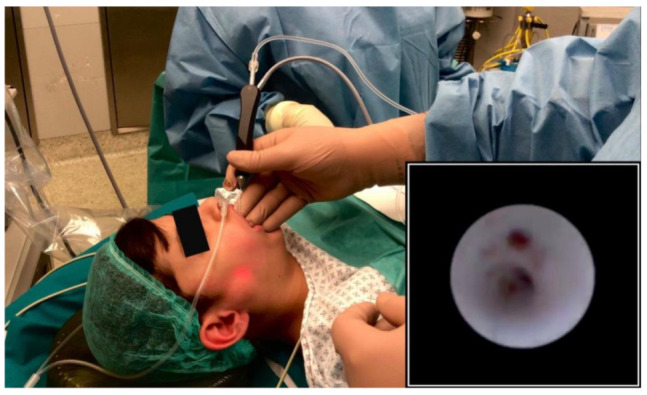
Transillumination of the light of the sialendoscope during parotid sialendoscopy under deep sedation; endoscopic view of the main parotid duct (picture in picture).

During sialendoscopy (Figure 1), diffuse sialodochitis with pale and whitish appearance of the duct and loss of vascular trauma was observed in five glands; sialodochitis was limited to the primary superior duct branch in the remaining three glands. A variable presence of mucous plugs was evidenced in all the patients. Multiple web-like stenoses of the mid third of the Stensen’s duct were observed in one patient and bilateral evidence of granulation tissue in the ductal wall was observed in one patient.

The median perceived symptom improvement was high (8.5/10, range 8–10). All parents and patients would repeat the procedure if needed, and would recommend it to others with similar symptoms. With regard to parental opinions about deep sedation, the mean evaluation of the child’s experience in the operatory room was very good (8.5/10, range 7–10).

At the three months follow-up, no new episodes of parotitis were reported on the treated glands; interestingly, a mild swelling of the contralateral untreated parotid gland was reported by one child. A telemedicine assessment performed in December 2020 (median follow-up 12, range 11–13 months) confirmed the lack of swelling recurrences of any treated gland in every patient.

The clinical, sialendoscopic and anesthesiologic data of patients and their relatives are described in Table 2 and Table 3.

## 4. Discussion

Deep sedation is a relatively new option for pediatric sialendoscopy in children with salivary disorders; among these, JRP represent the most frequent pathologic salivary condition. Deep sedation is considered a cost-effective third option, after local and general anesthesia, as it reduces unnecessary hospital stay and avoids invasive airway management [15].

To date, only local anesthesia has been proposed for pediatric sialendoscopy for salivary stones in few children older than 8 years [9,10]. Different pharmacological proposals are available to obtain an adequate sedation; a combination of propofol and fentanyl was proposed to our patients and parents due to the wide consensus on its effectiveness and safety for pediatric procedures. It was preferred to dexmedetomidine for its better outcome in emergence times, reported satisfaction and post-operative behavioral disturbances [16].

All the patients underwent a successful diagnostic and interventional sialendoscopy under deep sedation without adverse effects or post-operative complications along with a rapid hospital discharge. Sialendoscopy confirmed to be, as previously described [6], a valid and safe diagnostic and therapeutic tool for the management of JRP as none of the patients revealed new episodes of parotid swelling in the brief follow-up period; interestingly, a child referred a mild swelling of the contralateral asymptomatic parotid gland, thus confirming the hypothesis of an immune disorder as a causative event. Moreover, diagnostic sialendoscopy revealed typical endoscopic features of JRP, such as whitish appearance of the duct wall, mucous plugs, duct kinking and strictures, inflammatory granulation tissue [4]. With regard to sedation, we preferred to combine propofol to fentanyl to reduce discomfort, considering the possible painfulness of the procedure, and to allow a reduced propofol dosage. Moreover, continuous infusion for maintaining the target depth of sedation was preferred to intermittent bolus administration considering the risk of overshooting and consequent reduced or absent airway patency [17]. The post-operative pain management protocol in the study population (paracetamol i.v. 15 mg/kg max four times a day) was the same that we are used to adopting for treatment under general anesthesia, so the increased post-operative pain referred by patient n. 3 is probably not a consequence of failure of deep sedation that would not have occurred when conducting the treatment under general anesthesia. However, given that the same patient also experimented a full awakening event during the procedure, we can assume that the wake-up event triggered the more severe post-operative pain in this case. Therefore, it appears to be essential to maintain the deep sedation without any interruption in order to reduce the post-operative pain and make the procedure less uncomfortable for the patient.

Sialendoscopy is considered an advanced procedure in the training of residents in the USA [18] and so one of the procedures was done by a resident; no limitation by the relatively more aroused condition of the patient compared to general anesthesia was observed, which, conversely, could be a notable limitation during local anesthesia.

The average survey results showed a significant improvement of the symptoms and an overall very good experience about the deep sedation procedure. The worst scores were recorded on the perceived comfort of the environment of the operatory room before the procedure rather than on the technique itself, suggesting that future improvement may reside in developing a more calm and reassuring setting, i.e., possibly exploring an outpatient design, while maintaining full safety with constant anesthesiologic support and promptness of airway management if needed. Even if salivary gland irrigation with saline solution and steroid performed on outpatient basis can be taken into consideration for selected older children as alternative method to achieve symptomatic relief by inducing salivary ductal flushing, we do think that (diagnostic and interventional) sialendoscopy should be offered as a first line approach to both confirm diagnosis excluding other causes of pediatric recurrent salivary gland obstruction, and as a treatment modality.

Despite the low number of consecutive patients treated that does not allow us to draw any general conclusion, we promptly decided to show our experience given the positive preliminary results and the emergent COVID-19 pandemic that is locking down elective pediatric surgery; it could be a coincidence, but we recently described acute non suppurative parotitis as a possible manifestation of the COVID-19 disease spectrum [19].

## 5. Conclusions

We here present our preliminary positive experience with pediatric sialendoscopy performed under deep sedation with propofol and fentanyl for children with JRP. Despite these findings needing to be confirmed on a large case series, we think that this option can be considered among traditional therapeutic choices, especially in this moment, where the transition from general anesthesia to sedation for pediatric sialendoscopy could significantly reduce the risk of generating respiratory particle aerosols during airway maneuvers, in particular tracheal intubation [20].

## Figures and Tables

**Table 1:** Clinical and sialendoscopic data of children.

Patient No.	1	2	3	4	5	6	Mean
**Age**	6	8	11	11	13	12	10.2
**Sex**	F	M	F	M	F	M	-
**Gland side**	L/R	R	R	L/R	L	L	-
**Recurrences/years before** **sialendoscopy**	3	4	3	3	5	4	3.7
**Sialendoscopic features**	Diffuse sialodochitis; mucous plug	Segmental sialodochitis; web-like stenosis, mucous plug	Segmental sialodochitis; mucous plug	Diffuse sialodochitis; granulation mucosal tissue; mucous plug	Diffuse sialodochitis; mucous plug	Diffuse sialodochitis; mucous plug	-
**Immediate post-operative pain**	2	4	8	0	4	1	3.2
**3h post-operative pain**	0	0	6	0	2	0	1.3

**Table 2:** Symptoms improvement evaluation according to Gillespie et al. [13].

Questions	Patient Response	No. of Yes (Total: 6)	Mean (Range)	Median
(1) Do you continue to have trouble with your treated salivary gland?	Yes/No	1		
(2) Do you currently have swelling of the gland with meals?	Yes/No	0		
(3) Do you currently have swelling of the gland between meals?	Yes/No	0		
(4) How severe is the pain between 0 (no pain) and 10 (severe pain; need narcotics)?	Patient response 0–10		1.5 (0–6)	0
(5) Have you required further treatment for the salivary gland?	Patient response	1 (parotid massage)		
(7) Do you still have an intact (not surgically removed) gland?	Yes/No	6		
(8) Did the salivary endoscopy improve your symptoms?	Yes/No	6		
(9) How much improvement did you experience between 0 (no improvement) and 10 (gland feels normal)?	Patient response 0–10		9.2 (8–10)	8.5
(10) Would you choose to undergo the endoscopy again if you had the option?	Yes/No	6		
(11) Would you recommend the endoscopy to others with similar symptoms?	Yes/No	6		

**Table 3:** Anaesthesiologic procedure evaluation according to Iacobucci et al. [14].

**For the Parents (Answer 0–10)**	**Mean (Range)**	**Median**
(1) According to you, how satisfactory was the conversation between your child and the anaesthesiologist before the operation?	9.2 (8–10)	9.5
(2a) In the operating room, *before the operation*, how comfortable was this environment for you and your child?	6.8 (5–9)	7
(2b) In the operating room, *before the operation*, according to you, how satisfactory and gentle was the behaviour of the nursing staff?	9 (8–10)	9
(3a) In the operating room, *after the operation*, how sufficient was the observation period by the anaesthesiologist?	10	10
(3b) In the operating room, *after the operation*, how sufficient was the care provided by the nursing staff?	9.5 (8–10)	10
(4) In your opinion, will your child have a generally bad/negative memory of the operating room experience (0: bad, 10: very good)?	8.5 (7–10)	8.5
(5) If you should give an overall judgement on your child’s experience in our operating room would it be?	8.5 (7–10)	8.5
**For the Child (Answer Yes/No)**	**No. of Yes (Total: 6)**	
(1) Were you scared the day before the operation?	6	
(2) Did the anaesthesiologist make you feel calmer?	6	
(3) Was the operating room the way you thought?	0	
(4) Did it seem ugly to you? If yes, why:	1	
(4a) There were not nice people	0	
(4b) There were strange things	1	
(4c) There was something that scared me	1	
(4d) There were bad smells	0	
(4e) Other	0	
(5) When you fell asleep were you afraid?	3	
(5.1) Did the nurses make you feel better?	6	
(5.2) Were the nurses nice?	6	
(5.3) Was the doctor who made you go to sleep nice?	6	
(5.4) Did the doctor make you feel calm?	6	
(6) What was the thing that frightened you most of all?		
(6a) Your mummy was not there	3	
(6b) There were strangers all around you	3	
(6c) The fear of pain	4	
(6d) Other	0	

## Data Availability

Data is contained within the article.
